# Landscape genomic approach to detect selection signatures in locally adapted Brazilian swine genetic groups

**DOI:** 10.1002/ece3.3323

**Published:** 2017-10-12

**Authors:** Robson Jose Cesconeto, Stéphane Joost, Concepta Margaret McManus, Samuel Rezende Paiva, Jaime Araujo Cobuci, Jose Braccini

**Affiliations:** ^1^ Universidade Federal do Rio Grande do Sul Porto Alegre Rio Grande do Sul Brazil; ^2^ Laboratory of Geographic Information Systems (LASIG) School of Architecture, Civil and Environmental Engineering (ENAC) Ecole Polytechnique Fédérale de Lausanne (EPFL) Lausanne Switzerland; ^3^ Universidade de Brasília (UnB) Brasilia DF Brazil; ^4^ EMBRAPA Brasilia Brazil

**Keywords:** animal genetic resources, conservation genetics, molecular markers, population structure, *Sus scrofa*

## Abstract

Samples of 191 animals from 18 different Brazilian locally adapted swine genetic groups were genotyped using Illumina Porcine SNP60 BeadChip in order to identify selection signatures related to the monthly variation of Brazilian environmental variables. Using BayeScan software, 71 SNP markers were identified as F_ST_ outliers and 60 genotypes (58 markers) were found by Samβada software in 371 logistic models correlated with 112 environmental variables. Five markers were identified in both methods, with a Kappa value of 0.073 (95% CI: 0.011–0.134). The frequency of these markers indicated a clear north–south country division that reflects Brazilian environmental differences in temperature, solar radiation, and precipitation. Global spatial territory correlation for environmental variables corroborates this finding (average Moran's I = 0.89, range from 0.55 to 0.97). The distribution of alleles over the territory was not strongly correlated with the breed/genetic groups. These results are congruent with previous mtDNA studies and should be used to direct germplasm collection for the National gene bank.

## 1 INTRODUCTION

After the introduction to Brazil of Portuguese, Spanish, and Asian swine breeds in early 1500s, these animals spread throughout the Brazilian territory. Through equilibrium between evolutionary (crossbreeding, artificial selection, and mainly genetic drift) and local environment forces have originated several locally adapted swine breeds that one which have had sufficient time in the county for to be genetically adapted to the environment (Galal & Boyazoglu, 2001). Locally adapted swine breeds (e.g., Piau, Canastra) were used as an important source of meat and fat by farmers and the general population (Mariante, Castro, Albuquerque, Paiva, & Germano, 2003) until the 1970s, when changes in the market led to the introduction of North American (e.g., Duroc) and European (e.g., Landrace, Large White) breeds, specialized in meat production. At present, locally adapted breeds are present only on small farms, with low input levels.

These local breeds, results of a sum of economic, social, historical, and cultural factors, are a reservoir of genetic variability (Giovambattista et al. 2001) and, principally, source of traits selected and fixed mainly by the influence of the environment (Hall & Ruane, 1993). Mirkena et al. (2010) discuss genetic influence on disease tolerance/resistance in small ruminants and, in addition to other factors, cite advantages in increased fitness of locally adapted breeds, while Osman et al. *(* 2017) studied adaptability and suitability advantages of Egyptian local breeds and Traspov et al. (2016) highlighted the adaptation to local climate, feed, pathogens, and human preferences of Russian Belorussian, Kazakhstan, and Ukraine pig breeds. This adaptation to the environment can be evaluated by genomic analyses of areas of the genome that have been, or still are, under selection (Luikart, England, Tallmon, Jordan, & Taberlet, 2003; Storz, 2005; Vitalis, Gautier, Dawson, & Beaumont, 2014). These can be estimated using single nucleotide polymorphisms (SNPs) spread throughout the genome by theoretical populational F_ST_ outliers approach that are assumed to be signatures of natural selection (Lewontin & Krakauer 1973; Luikart et al., 2003; Joost et al., 2007; Lotterhos & Whitlock 2014). Signatures of selection were found by Ottoni et al. (2013) in pigs from archaeological sites, helping to understand some events of pig domestication in Western Eurasia, introgression of Asian genes in European pigs by human selection (Bosse, Megens, Frantz et al., 2014), and enables the identification of introgression among different breeds (Bosse, Megens, Madsen et al., 2014). These selection signatures can help us understand the complex relation between adapted swine genetic groups and the environment, as well as the process of adaptation of swine over the Brazilian territory and to overcome the challenges in swine management in a country with continental dimensions and different climatic conditions. In a constantly changing world, the identification of those signatures may be the key to promote more sustainable animal production, improving gains in productivity and welfare, as well as decreasing sanitary expenses with medication and management (Mirkena et al., 2010; Shabtay, 2015). They can also be used for branding of particular regional products (Herrero‐Medrano et al., 2013). In addition, these results might be an auxiliary tool to help the enrichment of National gene banks (Paiva, McManus, & Blackburn, 2016) and conservation programs as suggested by Nuijten et al. (2016) or Bosse et al. (2015), who show that management strategies to preserve the variation in managed populations can benefit by whole‐genome, high‐density, marker‐assisted methods.

The hypothesis of this study is that monthly variation from Brazilian environment by the years, as was seen with Vietinamese (Pham et al., 2014), American village pigs (Burgos‐Paz et al., 2012), and Chinese sheep (Yuan et al., 2017), influenced successful adaptation of swine in the Brazilian territory and left detectable signatures of natural selection. Understanding the influence of the environment on the process of allele selection can be useful to improve gains on small farms, preserve genetic variation from herds, and adaptation to world climatic changes.

To test this hypothesis, a medium SNP chip array of locally adapted swine breeds population, with animals sampled from over the main Brazilian regions, was used to identify selection signatures through FST Outliers approach.

## 2 MATERIALS AND METHODS

### 2.1 Sampling

The Brazilian territory is divided into five regions (each further divided into states) based on natural, cultural, social, and economic features. Despite the high mobility of swine, free movement of animals between states and regions is restricted by legal and sanity factors (Classical Swine Fever, African Swine Fever, Foot‐and‐Mouth disease, Aujeszky's disease). So, to capture high spatial representation of the environment and genetic territorial dispersion of the swine breeds over the Brazilian territory, the sample selection (Table 1 and S1) was structured with at least one sample from each Political Region (Figure S1). A total of 191 samples of nonrelated animals from 18 different swine genetic groups (13 locally adapted Brazilian swine genetic groups, four commercial or global breeds, and one group formed by crossbred animals) were randomly selected. All samples used in this experiment are deposited in Embrapa's Gene Bank (http://aleloanimal.cenargen.embrapa.br) located at Embrapa Genetic Resources and Biotechnology Center, Brasilia, DF. The samples from locally adapted Brazilian swine genetic groups were classified in accordance with a phenotypic description suggested by Viana (1956), Germano, Albuquerque, and Castro *(* 2002), and Mariante and Cavalcante (2006).

**Table 1:** Basic descriptive statistics of sampling (location and environment) of Brazilian locally adapted swine breeds

Region	State	Samples		Breed	Samples		Elevation		Temperature		Solar Radiation		Aridity		PETannual		Precipitation	
		Nº	%		Nº	%	Average	*SD*	Average	*SD*	Average	*SD*	Average	*SD*	Average	*SD*	Average	*SD*
N	PA	29	16.02	SMA	29	16.02	3.17	0.89	273.14	32.89	14.77	0.63	15619.83	18.68	1573.21	0.42	204.78	150.62
NE	BA	3	1.66	SPI	3	1.66	260	0	244.03	46.65	14.49	1.86	4443	0	1690	0	62.58	34.05
	PE	60	33.15	SBA	10	5.52	387.9	134.81	240.79	45.25	14.67	1.35	4168.3	1284.79	1638	123.64	55.94	48.63
				SCR	5	2.76	550	278.89	236.88	47.12	14.6	1.55	5007.6	2562.54	1654.6	70.63	67.77	67.29
				SCT	8	4.42	475.25	84.33	242.42	46.36	14.67	1.32	3983	611.41	1692.63	85.17	56.02	51.44
				SCTA	8	4.42	616.25	187.32	234.61	46.66	14.63	1.45	4705.38	1975.41	1650.75	107.82	63.91	63.75
				SDL	2	1.1	61	49.5	252.99	32.47	14.67	1.35	12692.5	313.25	1446.5	31.82	161.36	97.68
				SME	5	2.76	261	144.31	243.36	39.7	14.66	1.36	5699.8	2061.74	1543.8	200.49	70.87	50.84
				SMO	8	4.42	481.88	77.98	241.4	46.15	14.67	1.31	4094.5	562.62	1685.75	84.96	57.36	52.32
				SNI	7	3.87	511.57	91.09	239.68	45.79	14.67	1.32	4034.29	613.29	1671.57	72.38	56.04	53.19
				SPI	6	3.31	275	225.33	242.76	39.79	14.67	1.34	6644.17	3691.36	1528.17	133.1	81.99	71.27
				SUR	1	0.55	8	–	258.14	29.93	14.67	1.39	13025	–	1395	–	151.58	104.73
	PB	2	1.1	SCRI	2	1.1	557	0	217.58	39.3	14.7	1.25	8882	0	1467	0	108	60.93
CO	GO	27	14.92	SCB	3	1.66	1116	0	208.5	47.46	14.31	2.24	10976	0	1536	0	140.67	110.26
				SCR	3	1.66	1116	0	208.5	47.46	14.31	2.24	10976	0	1536	0	140.67	110.26
				SDL	3	1.66	959	0	216.31	48.85	14.32	2.22	9133	0	1581	0	120.42	96.74
				SLW	1	0.55	586	–	182.92	57.15	13.36	3.57	13095	–	1399	–	152.83	25.35
				SMO	1	0.55	1116	–	208.5	47.9	14.31	2.31	10976	–	1536	–	140.67	113.56
				SMT	5	2.76	1103.8	90.73	209.77	47.4	14.31	2.22	10227.6	839.62	1540.6	28.64	131.08	102.11
				SNI	3	1.66	895.33	258.45	221.36	49.5	14.43	1.98	8112.33	3487.86	1609	90.32	107.11	97.53
				SPI	6	3.31	1038.17	50.71	212.72	47.74	14.31	2.21	10008.5	1177.42	1554.83	26.83	129.63	103.03
				SRP	2	1.1	1116	0	208.5	47.57	14.31	2.25	10976	0	1536	0	140.67	111.06
	MS	18	9.94	SMT	18	9.94	98.83	5.75	258.45	48.74	14.1	2.55	7219.56	93.15	1696.83	11.77	102.1	61.3
	MT	3	1.66	SMT	3	1.66	122.67	2.89	261.86	51.05	14.28	2.28	6932.67	11.55	1785.67	0.58	103.17	68.62
SD	MG	3	1.66	SPI	1	0.55	658	–	205.64	53.72	13.96	2.85	8079	–	1526	–	102.42	81.82
				SPN	2	1.1	695	0	202.94	53.33	13.96	2.79	8197	0	1520	0	103.75	80.55
S	RS	14	7.73	SCR	3	1.66	140.67	30.02	176.17	56.42	12.87	3.91	10935.67	85.45	1271	5.2	115.83	19.53
				SME	1	0.55	155	–	175.58	56.88	12.87	4.02	10953	–	1269	–	115.92	20.01
				SMO	4	2.21	139.5	31	176.24	56.36	12.87	3.89	10927.75	50.5	1271.25	4.5	115.83	19.48
				SNI	5	2.76	83.8	9.09	184.11	52.56	12.93	3.83	11237.6	216.23	1244.8	18.57	116.55	17.37
				SPI	1	0.55	89	–	185.25	52.12	12.95	3.96	11351	–	1236	–	116.92	17.31
	SC	22	12.15	SCB	1	0.55	401	–	181.58	47.81	13.27	3.66	12648	–	1216	–	128.17	34.67
				SDC	4	2.21	586	0	182.92	56.55	13.36	3.45	13095	0	1399	0	152.83	24.53
				SDL	7	3.87	433.86	103.93	184.52	52.09	13.3	3.49	12064.29	704.11	1324.71	50.75	133.96	33.34
				SLW	2	1.1	586	0	182.92	56.75	13.36	3.49	13095	0	1399	0	152.83	24.79
				SMO	8	4.42	436	182.4	185.06	51.93	13.32	3.48	12811.5	316.36	1305.5	100	139.46	32.02
Brazil		181	100		181	100	398.28	352.05	231.25	55.56	14.25	2.3	9233.5	4201.14	1550.34	156.12	116.41	97.42

*N*, number of samples; SD, standard deviation; PETannual, annual potential evapotranspiration.

Region: N, north; NE, northeast; CO, midwest, *SD*, southeast; S, South; State: PA, Pará; BA, Bahia; PE, Pernambuco; PB, Paraíba; GO, Goiás; MS, Mato Grosso do Sul; MT, Mato Grosso; MG, Minas Gerais; RS, Rio Grande do Sul; SC, Santa Catarina; Breed: SBA, Baé; SCB, Casco de Burro; SCR, Caruncho; SMEc, Crioulo; SCT, Canastra; SCTA, Canastrão; SDC, Duroc; SLD, Landrace; SLW, Large Withe; SMA, Marajó; SME, Mestiço; SMO, Moura; SMT, Monteiro; SNI, Nilo; SPI, Piau; SPN, Pietran; SRP, Rabo de Peixe.

### 2.2 Genotyping and quality control

The DNA samples were genotyped with the Illumina Porcine SNP60 BeadChip v2. To eliminate SNPs with low‐quality identification, monomorphic markers, SNPs recently fixed in the populations and lower informative samples that could generate as false‐positive selection signatures as bias, quality control of raw data (191 samples and 61,565 SNP markers) was performed with SNP & Variation Suite v8.x (Golden Helix, Inc., Bozeman, MT, USA 2015). We chose parameter thresholds as reported in literature (Bosse, Megens, Madsen et al., 2014; Burgos‐Paz et al., 2012; Traspov et al., 2016) that eliminated low‐quality SNPs/Samples but preserve a maximum number of samples: minimal individual genotype call rate of 90% that excluded 11 samples; 95% call rate and 0.05% minor allele frequency (MAF) for the markers when 21,605 SNPs were excluded. Additional linkage disequilibrium (LD) pruning was performed using a window size = 50, window increment = 5, and r2 threshold = 0.05, which eliminated a further 11,646 SNPs. The final data had 28,860 SNP markers with an SNP density of 1/87,026 kb.

### 2.3 Environmental variables

The environmental variables from the Brazilian territory were obtained at the World ClimProject (http://www.worldclim.org/), GTOPO30 (https://lta.cr.usgs.gov/GTOPO30), and Harvest Choice (http://harvestchoice.org/) and 30‐s geographical information system (GIS) layer using Qgis v2.6 (QGIS Development Team 2009). Monthly maximum, average and minimum temperature, annual average and median for maximum, average and minimum temperature, seasonal averages and medians for maximum, average and minimum temperature, monthly solar radiation, annual average and median solar radiation, seasonal averages and medians solar radiation, monthly precipitation, annual average and median precipitation seasonal averages and medians precipitation, 19 bioclimatic variables (BIO1‐19), elevation, PETannual (annual potential evapotranspiration), and Aridity (ratio of precipitation to PET) were obtained for each sample from this layer (Table S2). As monthly and season variation in climatic condition has interfere in reproductive performance (De Rensis, Ziecik, & Kirkwood, 2017; Petrocelli, Batista, & Gosálvez, 2015; Prunier, Quesnel, de Bragança, & Kermabon, 1996), pulmonary disease (Eze et al., 2015; Gao, Xiao, Qin, Cao, & Wang, 2016) piglet early survey (Berger et al., 2007; Iida & Koketsu, 2014) in all stages of life (Ross et al., 2015; Wildt, Riegle, & Dukelow, 1975), including intrauterine development (Johnson et al., 2013, 2015a), we used the environmental variables in an exploratory approach, to identify the influence of each explanatory variable on the allele frequencies.

### 2.4 Relationship between samples

The individual and populational levels of expected heterozygosity (H_e_) and observed heterozygosity (H_o_) were computed by the SNP & Variation Suite v8.x (Golden Helix, Inc., Bozeman, MT, USA 2015) and used to calculate the inbreeding coefficient (*F*_IS_), to identify sources of genetic variance by means of analysis of molecular variance (AMOVA) between and within the geopolitical groups (samples within region), as well as between native and commercial breeds (Tables S3 and S4), performed in Arlequin V 3.5.2.2 (Excoffier, Laval, & Stefan, 2005). Population structure analysis was performed in STRUCTURE (Pritchard, Stephens, & Donnelly, 2000), and a number of groups identified by second‐order rate of change of the likelihood (∆*K*) (Evanno, Regnaut, & Goudet, 2005). The discontinuity of genetic composition was evaluated using a Mantel test between the Euclidian geographical distance and the genetic distance *F*_ST_/(1−*F*_ST_) was calculated in PASSaGE (Rosenberg & Anderson, 2011).

### 2.5 Signatures of selection and outlier detection

Loci with high or low allelic differentiation in relation to the expected neutrality, from the 28,860 SNPs in final data, were used as an indication of selection (Hoffmann & Willi, 2008) and were tested by two different methodologies of outlier identification.

BayeScan software V 2.1 (Foll & Gaggiotti, 2008) used a Bayesian approach via Markov Chain Monte Carlo (MCMC), assuming a prior Dirichlet distribution of alleles within populations and a hierarchical Bayesian model. The program calculates posterior odds, from the posterior probability of the models, with and without selection on a locus, using the proportion of loci with a strong increase in *F*_ST_ relative to other loci among the MCMC outputs of its simulations (Beaumont & Balding, 2004). The software was set up with 5,000 burn‐in interactions, followed by 10,000 interactions with thinning interval of 10. Convergence was verified using CODA package for R (Plummer, Best, Cowles, & Vines, 2006) with critical values of −1.96 > *z* > +1.96. A second analysis was performed using the software Samβada (Joost et al., 2007; Stucki et al., 2014) that used logistic regression models to determine the probability of allele presence/absence in a specific environment. The models were considered significant when the G Score and Wald Score were significant at α = 0.01 threshold with a Bonferroni correction. The G Score can be defined as the ratio between maximum log likelihood of model with the presence of the independent variable and the maximum log likelihood of model without independent variable, or as the independent variable affects in the log likelihood model. The Wald Score tests if goodness of fit is affected when the independent variable is removed from the model. Using the FREQ procedure (Proc FREQ) of SAS v9.3 (SAS Institute Inc. 2011), the agreement between the two methods was evaluated through the Kappa index. The Kappa index is a measure of interrater agreement, between two or more methods: When the observed agreement exceeds chance agreement, kappa is positive, with its magnitude reflecting the strength of agreement. Gene annotations within candidate regions were obtained using the data provided by Ensembl (Cunningham et al., 2015) and NCBI (http://www.ncbi.nlm.nih.gov). To explore the linkage disequilibrium (LD) of selection signatures detected with other FST outliers and with nearby genes, we calculated the LD from these markers using Plink software (Purcell, 2014).

To measure the degree of spatial association for marker signaled as F_ST_ outliers by both methods, the Global spatial autocorrelation (Moran's I) was calculated. Moran's I describes the autocorrelation between the values of a variable in a certain location with the values of this same variable in a neighboring location (Druck, Carvalho, Câmara, & Monteiro, 2004), with null hypothesis being that there is no spatial clustering.

## 3 RESULTS

Molecular variance analysis among states grouped into regions (Table S3) showed 93.35% of the genetic variance was contained within states and only 0.87% among regions. The genetic variance among a group of animals from commercial breeds and a group of locally adapted genetic groups (Table S4) showed individual variance (81.85%) was larger than variance between groups (3.66%) or from individuals within groups (8.27%).

The *F*_IS_ (Table S5) varied from −0051 to 0.642 (within breeds) showing that inbreeding levels of the naturalized breeds vary considerably within subpopulations, and was consistent with populational structure found in STRUCTURE analyses. Among locally adapted breeds, the Monteiro breed showed the highest average values of *F*_IS_ (μ = 0.289), which indicates low genetic differentiation between individuals within the herds of this breed. Within regions, the populations from the State of Mato Grosso do Sul showed higher inbreeding levels (μ = 0.296), while the animals from the State of Paraiba revealed a tendency toward excess of heterozygotes (μ = −0.001).

The theoretical number of actual populations using the genetic frequencies of the loci to infer the influence of the genetic groups on the composition and number of populations (Figure S2) was determined. The individuals were adequately allocated inside their original population by means of a *K* equal to the number of sampled breeds (*K* = 18). But, using the second‐order rate of change of the likelihood (Δ*K*), the number of groups was reduced to seven (*k* = 7).

**Figure 1:**
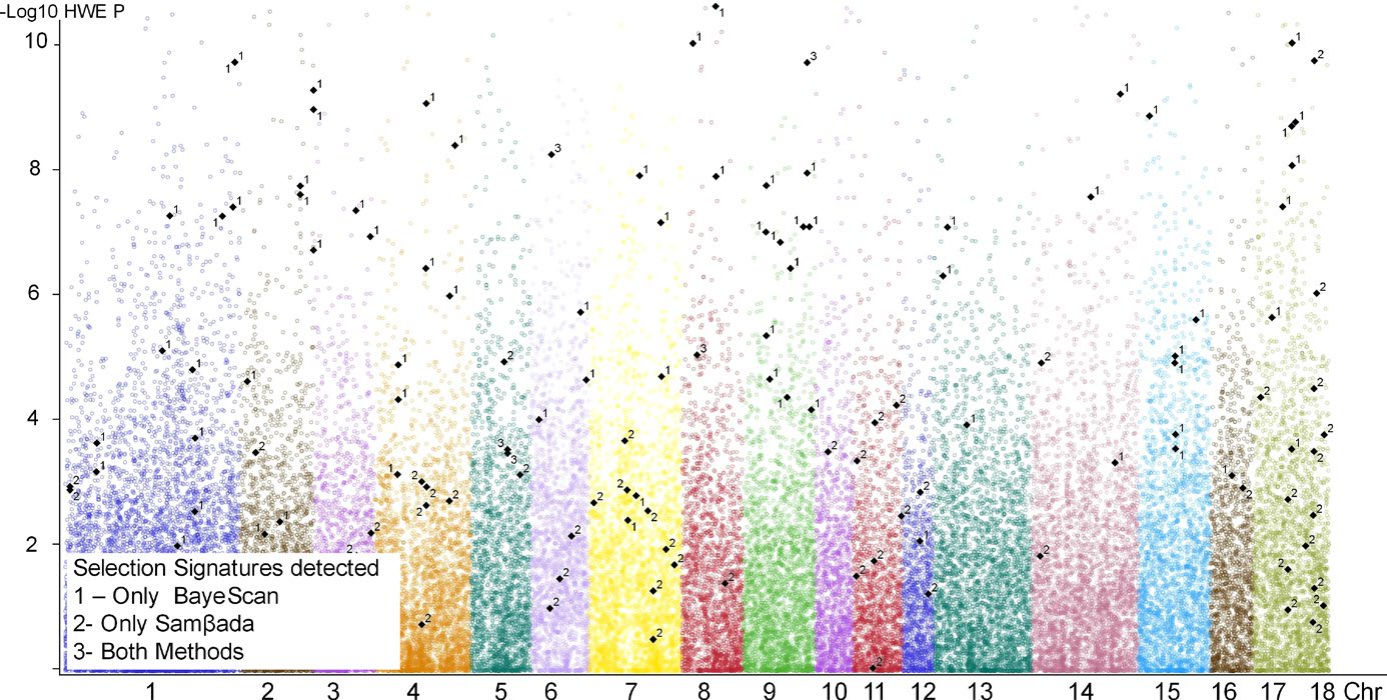
Genome position of *F*_ST_ Outliers detected by SAmβada (1), BayeScan (2), and through both methods (3)

**Figure 2:**
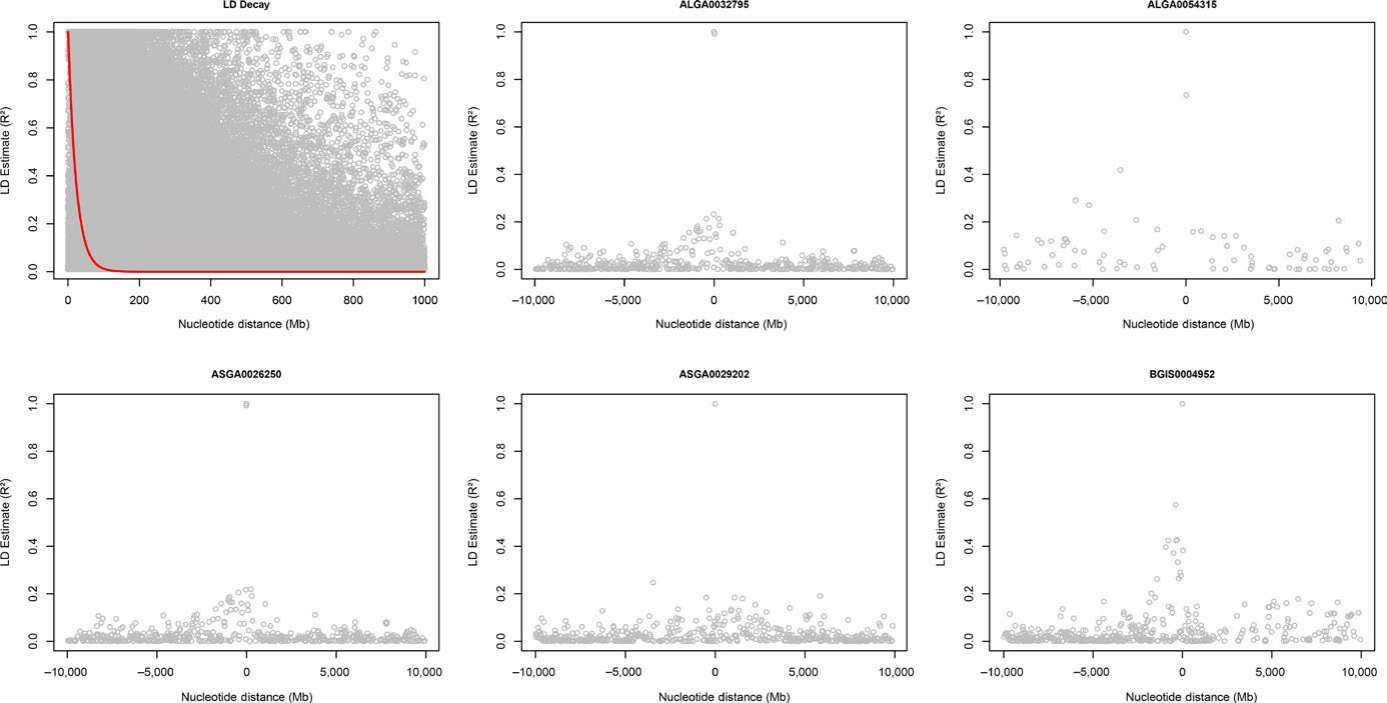
Linkage disequilibrium decay and linkage disequilibrium up to 1,000 Mega bases (Mb) around the markers identified as selection signatures by Samβada/BayeScan

BayeScan software identified 71 SNP markers as F_ST_ outliers, while Samβada software identified 60 genotypes (from 58 SNP markers) in 371 univariate logistic models, using 112 environmental variables. No multivariate model was significant at α=0.05. The markers MARC0021990; ASGA0033717; MARC0007678 were responsible for 42% of all models generated by Samβada (Figure S3). Five markers, associated with different environmental conditions (Table 2), were found using both methods (ALGA0032795; ALGA0054315; ASGA0026250; ASGA0029202; BGIS0004952) with Kappa 0.073 (95% CI: 0.011–0.134) located in regions of the genome with the presence of several assumed F_ST_ outliers (Figure 1). The linkage disequilibrium (Figure 2) of markers identified as signatures of selection suggested they could be associated with nearby genes (Table 3) responsible for intracellular transport, immune response, cell respiration, and related to the circulatory system, probably as a physiological response to cellular stress (Table 3). The marker ASGA0029202 was associated with precipitation and thermal amplitude and is near (±0.2 Mb) the CDH2 gene that has an effect on the formation of blood vessels, while the marker MARC0021990 (responsible for 20% of the models) was close to (±0.27 Mb) the gene CYP7B1 which is involved, among other functions, with cofactor HEME, suggesting indirect evidence of importance of circulatory system on genetic adaptation to fluctuation on temperature, Bio18 (precipitation of warmest quarter), and solar radiation in the Brazilian territory (Table 3).

**Table 2:** Samβada output to environmental association to markers detected as signatures of selection in both methods

Marker	Env_1	Loglikelihood	Gscore	WaldScore	Beta_0	Beta_1
ALGA0032795	TMinoutMedinan	−89.93	50.10	38.44	3.85	−0.03
ALGA0032795	TMINMai	−89.93	50.10	38.44	3.85	−0.03
ALGA0054315	TMAXAbr	−88.34	50.37	38.83	11.20	−0.04
ASGA0026250	TMinoutMedinan	−88.46	51.60	39.19	3.92	−0.03
ASGA0026250	TMINMai	−88.46	51.60	39.19	3.92	−0.03
ASGA0026250	TMinoutMed	−88.62	51.28	38.71	4.17	−0.03
ASGA0026250	TMINAbr	−89.15	50.22	38.57	5.26	−0.03
ASGA0029202	Bio18	−99.32	49.84	39.36	−2.41	0.01
BGIS0004952	Bio18	−94.83	60.58	45.77	−2.87	0.01
BGIS0004952	RadSolPrimMed	−95.64	58.96	41.26	−36.92	2.30
BGIS0004952	RadSolPrimMediana	−95.75	58.75	42.09	−30.26	1.87
BGIS0004952	RadSolNov	−95.87	58.51	41.34	−30.74	1.90
BGIS0004952	RadSolJAn	−96.23	57.80	41.04	−25.05	1.54
BGIS0004952	RadSolDez	−96.57	57.11	40.59	−20.93	1.29

Env_1, environment; TMinoutMedinan, median minimal temperature in autumn; TMINMai, minimal temperature in May; TMAXAbr, maximum temperature in April; TMinoutMed, average minimal temperature in autumn; TMINAbr, minimum temperature in April; Bio18, precipitation of warmest quarter; RadSolPrimMed, average of solar radiation to spring; RadSolPrimMediana, median of solar radiation to spring; RadSolNov, solar radiation in November; RadSolJAn, solar radiation January; RadSolDez, solar radiation December.

**Table 3:** Position in genome, distance from nearest genes, and biological function of these genes of markers identified as selection signatures in Brazilian locally adapted swine breeds

Marker	Crh	Most severe consequence	Gene	Markers is between (in reverse strand)			
				Gene (Distance from SNP)	Function	Gene (Distance from SNP)	Function
ALGA0032795	5	Intergenic		ENSSSCG00000024523 (±0.003 Mb)	Transmembrane transporter activity	ENSSSCG00000000783 (±0.3 Mb)	Glucose transmembrane transporter activity
ALGA0054315	9	Upstream gene	ENSSSCG00000015405		Cell surface receptor signaling pathway/immune response/		
		Intergenic		ENSSSCG00000015405 (±0.8 Mb)	Cell surface receptor signaling pathwaySource: InterPro	ENSSSCG00000015406 (±0.08 Mb)	Immune response
ASGA0026250	5	Intergenic		ENSSSCG00000024523 (±0.05 Mb)	Glucose transmembrane transporter activity	ENSSSCG00000000783 (±0.1 Mb)	Glucose transmembrane transporter activity/sugar:proton symporter activity.
ASGA0029202	6	Intergenic		ENSSSCG00000003720 (±0.6 Mb)	Transporter activity	ENSSSCG00000003722 (±0.2 Mb)	Calcium ion binding (blood vasal morphogenesis….)
BGIS0004952	8	Intergenic	COMMD8				
MARC0021990	4	Intergenic		ENSSSCG00000019505 (±0.1 Mb)	RNA genes	ENSSSCG00000019140 (±0.0075 Mb)	RNA genes
				ENSSSCG00000022092/ENSSSCG00000006222 (±0.2 Mb)	Iron (heme axial ligand)/RNA polymerase II transcription factor activity, sequence‐specific DNA binding		
ASGA0033717	7	Intron	ENSSSCG00000001771		Rho guanyl‐nucleotide exchange factor activity		
MARC0007678	3	Intergenic		ENSSSCG00000008605 (±0.4 Mb)	Uncharacterized protein	ENSSSCG00000008606 (±0.0001 Mb)	metal ion binding

Source:www.ensembl.org and http://www.ncbi.nlm.nih.gov/

Mb, mega bases.

**Figure 3:**
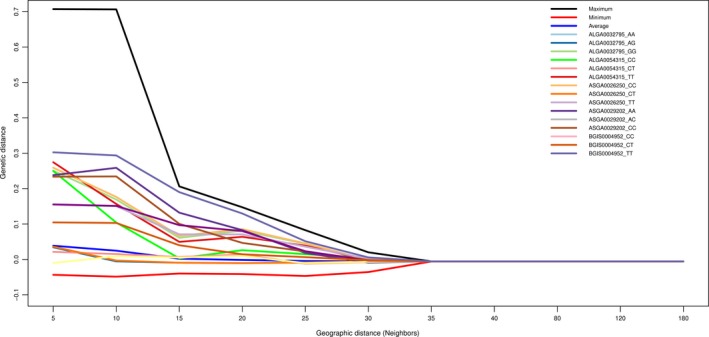
Moran′s I correlogram from genotypes of the markers identified as selection signatures in Brazilian locally adapted swine breeds by BayeScan and Samβada. Maximum, minimum, and average from all markers

Global spatial correlation for environmental variables was high, with 5 (five) neighbor windows (average Moran's I = 0.89, from 0.55 to 0.97), and reaching close to zero with 15 (fifteen) neighbor windows. The highest value for Moran′s I was associated with solar radiation in the summer months. The selection signal markers have had a high global spatial correlation between 5 and 10 neighbors and present a rapid decrease to zero with 35 neighbors (Figure 3). With five neighbors, the maximum local I was 0.7072 from marker CASI0001257 and the smallest was −0.04346 from marker ASGA0002592 (Figure 3).

**Figure 4:**
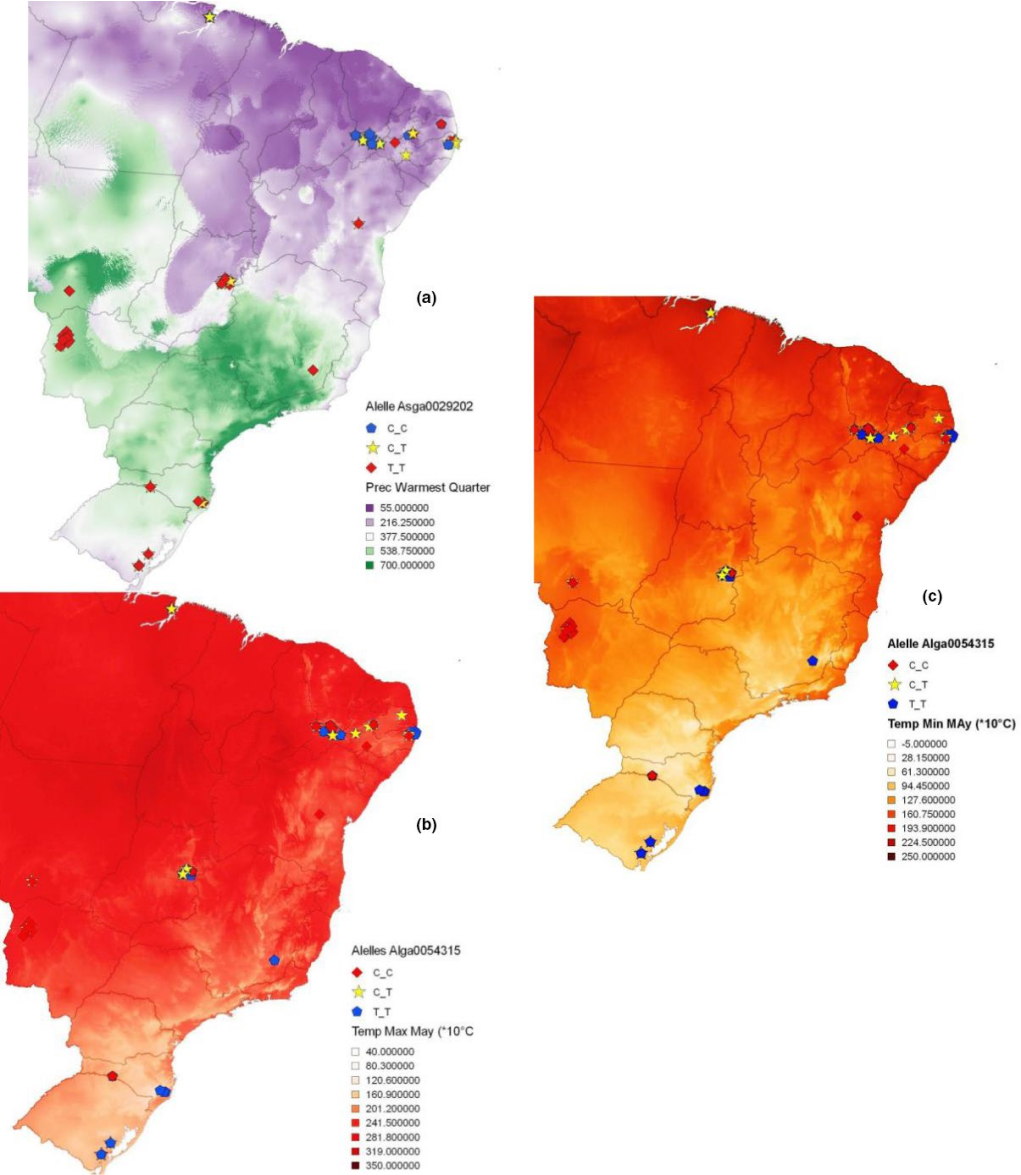
Maps of sampling points and genotype distribution maps from markers identified as selection signatures by Samβada/BayeScan. (a) Marker Asga0029202 in Bio18 layer (precipitation of Warmest Quarter). (b) Marker Alga0054315 in Minimum Temperature in May layer and (c) Marker Alga0054315 in Maximum Temperature in May Layer

For these five markers, considered selection signatures in BayeScan and Samβada, we found a nucleus of homozygotes in the neighborhood, but only with up to 30 neighbors. A regionalization of these markers was observed around a nucleus of climatic variation (Figure 4), with a loss of influence when geographical distance between samples was increased, or when distancing from the climatic influence center was decreased.

The Mantel test between individual pairwise genetic distances Fst/(1‐Fst) and individual geographical distance had correlation coefficient of *r* = 0.02 (*t* = 0.58, *p*(r) > 1e^−05^, with 99,999 replications).

## 4 DISCUSSION

The evolution and adaptation of pigs are subject to environmental influences, as has been observed in humans (Storz 2010), humans and cattle (Beja‐Pereira et al., 2003), fish (Nielsen et al., 2009), and other species (Manthey & Moyle, 2015). The genetic variability and population structure found were similar to other populations (Boitard et al., 2010; Burgos‐Paz et al., 2012) and other approaches (Sollero et al., 2009). A history of geographical isolation from the other breeds was in agreement with highest F_IS_ values presented by the Monteiro breed: a breed raised only in the states of Mato Grosso do Sul and Mato Grosso.

The small number of groups suggested by STRUCTURE, similar to that found by Sollero et al. (2009) working with Brazilian pigs and microsatellites, reveals that the breeds share alleles, possibly caused by interracial mating, including commercial breeds as found by Traspov et al. (2016), a common behavior carried out by small Brazilian farmers. Brazilian pigs have a high genetic variability, similar to that among locally adapted breeds found for Vietnamese (Pham et al., 2014), Indian (De et al., 2013), and Colombian pigs (Burgos‐Paz et al., 2012). This genetic diversity could be connected with the environment through years of selection, leaving marks on the swine genome. There are many different methodologies for the detection of genetic markers or genomic regions under influence of natural selection, and one of these approaches is the identification of populational theoretical F_ST_ outliers. The use of georeferenced environmental data associated with F_ST_ outliers helps in the understanding of the evolutionary process and the influence of the environment on this process.

For this work, we used two methods to detect Outliers in *F*_ST_. According to Pérez‐Figueroa, García‐Pereira, Saura, Rolán‐Alvarez, and Caballero (2010), BayeScan's algorithm under neutral hypothesis admits less than 1% of false discoveries, when we assume the Direchlet distribution and that the population has a neutral structure. Those presuppositions on the distribution and structure may become biased due to the existence of more than one sample within the population, or when individuals share a common ancestor in the recent past (Lotterhos & Whitlock 2014). Feng, Jiang, and Fan (2015) argue that some BayeScan configurations can affect the proportion and the direction of the markers in selection. This kind of bias does not occur with Samβada, because it translates samples in alleles frequencies associated with ambient data and uses these outliers to calculate logistic regression, which explains allele presence in a specific environment (Stucki et al., 2014). As the Samβada algorithm is based on individual and local levels, taking into consideration the *p*‐value after Bonferroni correction to determinate the significance of the models, the probability of mistakenly considering significant an association between marker and environmental variables decreases (Stucki, 2014; Stucki et al., 2014).

The rates of spatial autocorrelation (Figure 3) showed that the 5 to 10 closest neighbors tend to have high spatial autocorrelation among each other. This behavior was possibly motivated by the habit of farmers interchanging sires and dams, trying to maintain inbreeding at low levels (Favero & de Figueiredo, 2009; Gama et al., 2013). The probability of genetic similarity at a distance higher than 10 neighbors decreases, and this might be related to the limited dispersion due to sanitary legislation within the country for swine species, as well the market organization.

The pattern of spatial distribution of the genotypes, identified as selection markers (Figure 4), associated with environmental conditions such as temperature, solar radiation, and BIO18‐ precipitation of the warmest quarter (Table 2), during some periods in the year, shows adaptive selection linked to seasonality. The genotypic frequency of these signatures of selection divides the territory into two regions (Table 4), one in the north where we have predominantly the occurrence of one of the genotypes and the other to the south where the alternative genotype occurs. According to Nimer (1979), these two regions are identified by different climates: the north shows “equatorial,” “tropical,” and “northeast occidental tropical” climates; the south shows “temperate” and “central Brazil tropical” climates.

**Table 4:** Frequencies of genotypes from markers detected as selection signature according to Brazilian political regions

Marker	ALGA0032795			ALGA0054315			ASGA0026250			ASGA0029202			BGIS0004952		
Marker Genotype	GG (%)	GA (%)	AA (%)	TT (%)	CT (%)	CC (%)	TT (%)	CT (%)	CC (%)	AA (%)	AC (%)	CC (%)	TT (%)	CT (%)	CC (%)
Region															
North	2	14	35	2	17	26	35	13	2	2	20	50	4	35	21
Northeast	20	39	42	26	49	30	42	40	19	27	43	42	20	33	76
Midwest	28	32	20	24	19	34	20	31	29	39	20	3	41	17	3
Southeast	8	2	0	3	0	5	0	1	8	5	2	0	5	2	0
South	42	14	4	45	15	4	4	13	42	28	14	6	30	13	0
Genotype frequency Total	100	100	100	100	100	100	100	100	100	100	100	100	100	100	100

Although the markers MARC0021990; ASGA0033717; MARC0007678 were responsible for a high number of significant models identified in Samβada, we did not find any significant multivariate model (Figure S3). When one marker is linked to some environmental variables, this infers that many evolutionary steps within the environment, throughout the year, influence the presence of markers. Despite only univariate models being found, there were associations between these alleles and the variation of temperature throughout the year, but not among the seasons as discussed by Martyn Plummer et al. (2006). The environmental temperature is closely linked to welfare (Lee & Phillips, 1948) and animal productivity (Collier & Gebremedhin, 2015), affecting pigs in all stages of life (Ross et al., 2015; Wildt et al., 1975), including intrauterine development, with consequences in the postnatal development of animals (Johnson et al., 2013, 2015a). The significant models found by Samβada for mean diurnal range (BIO2) associated with the marker ALGA0012967 in an intronic region of the LGR4 gene, which directly influences the testicular development and spermatogenesis, were in accordance with Petrocelli et al. (2015) who reported seasonal variation of seminal quality parameters affecting the reproductive performance of females. Once the survival and adaptation of the species in the environment are limited by reproductive success from individuals, and knowing when environmental conditions such as temperature and humidity are outside thermal comfort limits, we can see physiological alterations leading to reproductive failure in females (Nteeba et al. 2015) and males (Flowers, 2015; Wettemann & Bazer, 1985).

Ai, Huang, and Ren (2013), working with Chinese pigs in Tibet, and Burgos‐Paz et al. (2012) with American pigs, found selection signatures correlated with the extremes of environmental conditions (high‐altitude adaptation), linked to altitude and circulatory system, respectively. Different from these authors, we found selection signatures for variation in temperature, radiation solar, and BIO18 (Table 2; Figure 4). The identification of selection signatures helps us to understand the relationship between climate and adaptive genetic variation, informing the conservation of both putatively neutral and adaptive components of genetic diversity (Bradbury, Smithson, & Krauss, 2013) across a dynamic and heterogeneous unpredictable landscape. Selection signatures from autochthone breeds may be a tool to improve livestock production through changes in the frequencies of these alleles in commercial herds, improving the adaptation in different environments. This is important in a world marked by environmental change that acts by altering the composition of the community and shifting range boundaries, phenology, genetic diversity, and genetic structure of organisms (Manel et al., 2012), probably imposing strong selection pressures on traits important for fitness (Gienapp, Teplitsky, Alho, Mills, & Merilä, 2008).

## 5 CONCLUSION

Allele frequency of markers from Brazilian locally adapted swine breeds was seen to be under the influence of environmental conditions showing evidence of footprints of divergent selection in at least 8 (eight) SNP markers, associated with temperature, solar radiation, and BIO18 linked with intracellular activity and circulatory system and were considered important for species adaptation.

The distribution of SNP alleles over the Brazilian territory demonstrates a clear north–south orientation, dividing the country into two distinct regions according to climatic conditions, drier and sunnier in the North and wetter and colder in the South. This information on selection signature distribution across Brazilian territory could be included in programs of assisted selectin using genetic markers, helping farmer through easier management of animals selected for adaptive characteristics. In the same way, the markers could be used to direct animals for more suitable regions according to their genotype in both traditional husbandry situations as well as genetic resource conservation programs.

## CONFLICT OF INTEREST

None declared.

## DATA ACCESSIBILITY

Genetic data deposited at Animal GRIN/Alelo Animal. It is available in http://nrrc.ars.usda.gov/A-GRIN/genomic_account_page?record_source=US.

## AUTHORS CONTRIBUTION

All authors enrolled have had equal contribution to the work presented in this paper, since the acquisition of data, until final approval of the version to be published.
