# Supplementary material for: Landscape genomic approach to detect selection signatures in locally adapted Brazilian swine genetic groups
Source: Ecol Evol. 2017 Oct 12;7(22):9544–56. doi: 10.1002/ece3.3323 (PMC5696410; doi:10.1002/ece3.3323)

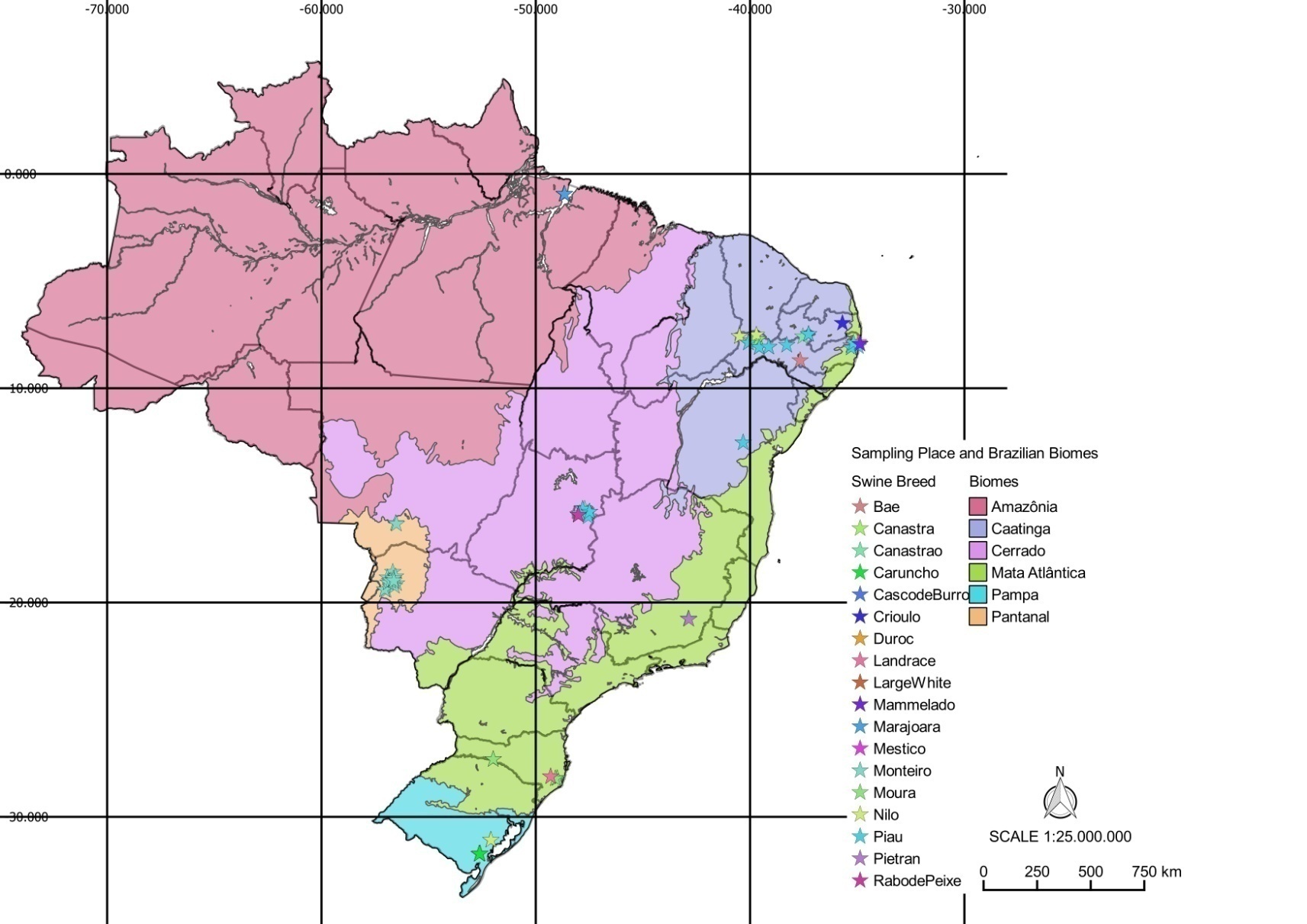
 Figure S1: Sampling location for Brazilian locally adapted swine breeds with biomes and states on the Brazilian territory.


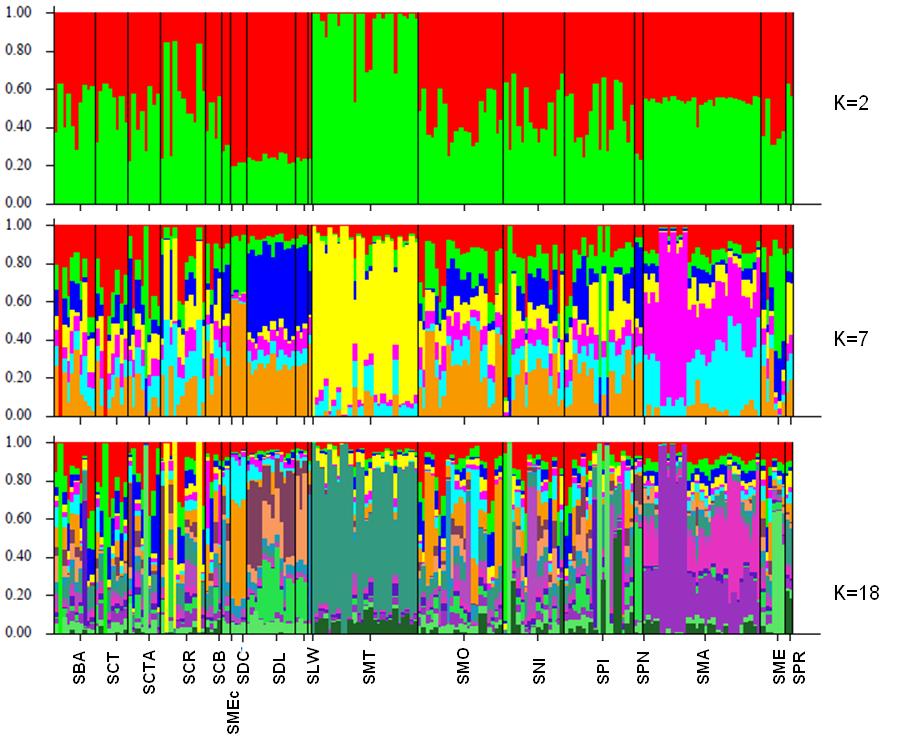
Figure S2: Population structure assessed by STRUCTURE. Bar plot. Each individual is represented by a vertical bar, often partitioned into colored segments with the length of each segment representing the proportion of the individual’s genome from K = 2, 7, or 18 populations

SBA= Baé; SCB= Casco de Burro; SCR= Caruncho; SMEc= Crioulo; SCT= Canastra; SCTA= Canastrao; SDC= Duroc; SLD= Landrace; SLW= Large Withe; SMA= Marajo; SME= Mestiço; SMO= Moura; SMT= Monteiro; SNI= Nilo; SPI= Piau; SPN= Pietran; SRP= Rabo de Peixe

Figure S3: Frequencies of number of model for each marker detected as selection signature in Samβada
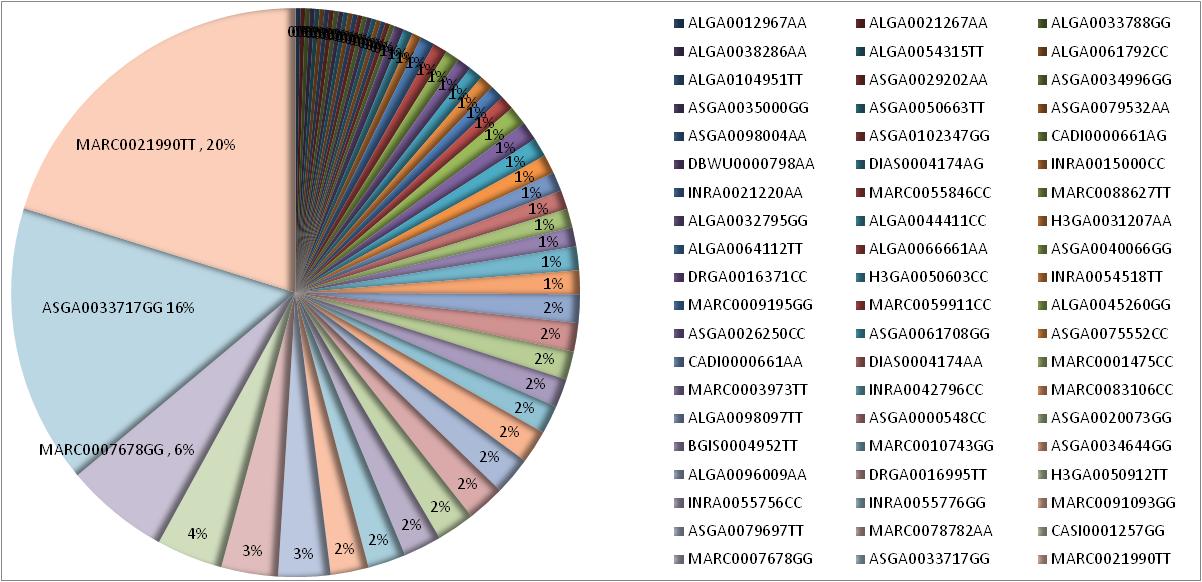

Supplement: Supplementary file 1 [file ECE3-7-9544-s001.docx]
