# Supplementary material for: Landscape genomic approach to detect selection signatures in locally adapted Brazilian swine genetic groups
Source: Ecol Evol. 2017 Oct 12;7(22):9544–56. doi: 10.1002/ece3.3323 (PMC5696410; doi:10.1002/ece3.3323)
Supplement: Supplementary file 2 [file ECE3-7-9544-s002.docx]

Table S1: Geolocation of Samples from Locally Adapted Brazilian Swine Genetic Groups

| Political region | State | Breed | Longitude | Latitude | Elevation |
| --- | --- | --- | --- | --- | --- |
| MidWest | Goiás | Caruncho | -48.013 | -15.9126 | 1116 |
|  |  | Caruncho | -48.013 | -15.9126 | 1116 |
|  |  | Caruncho | -39.578 | -8.09019 | 1116 |
|  |  | Casco de Burro | -48.013 | -15.9126 | 1116 |
|  |  | Casco de Burro | -48.013 | -15.9126 | 1116 |
|  |  | Casco de Burro | -48.013 | -15.9126 | 1116 |
|  |  | Landrace | -47.4928 | -15.7635 | 959 |
|  |  | Landrace | -47.4928 | -15.7635 | 959 |
|  |  | Landrace | -47.4928 | -15.7635 | 959 |
|  |  | Large White | -47.4928 | -15.7635 | 586 |
|  |  | Monteiro | -47.5706 | -15.8195 | 959 |
|  |  | Monteiro | -48.013 | -15.9126 | 1116 |
|  |  | Monteiro | -48.013 | -15.9126 | 1116 |
|  |  | Monteiro | -47.7194 | -15.6333 | 1117 |
|  |  | Monteiro | -48.0283 | -15.8797 | 1211 |
|  |  | Moura | -48.013 | -15.9126 | 1116 |
|  |  | Nilo | -40.4968 | -7.57808 | 611 |
|  |  | Nilo | -47.4928 | -15.7635 | 959 |
|  |  | Nilo | -48.013 | -15.9126 | 1116 |
|  |  | Piau | -47.4928 | -15.7635 | 959 |
|  |  | Piau | -47.5576 | -15.9901 | 1019 |
|  |  | Piau | -47.9574 | -15.8435 | 1041 |
|  |  | Piau | -47.8167 | -15.6334 | 1047 |
|  |  | Piau | -47.8167 | -15.6334 | 1047 |
|  |  | Piau | -48.013 | -15.9126 | 1116 |
|  |  | Rabo de Peixe | -48.013 | -15.9126 | 1116 |
|  |  | Rabo de Peixe | -48.013 | -15.9126 | 1116 |
|  | Mato Grosso | Monteiro | -56.5414 | -16.3011 | 121 |
|  |  | Monteiro | -56.5414 | -16.3011 | 121 |
|  |  | Monteiro | -56.501 | -16.3327 | 126 |
|  | Mato Grosso do Sul | Monteiro | -56.9858 | -19.4288 | 91 |
|  |  | Monteiro | -57.0568 | -19.3976 | 93 |
|  |  | Monteiro | -57.0568 | -19.3976 | 93 |
|  |  | Monteiro | -57.0568 | -19.3976 | 93 |
|  |  | Monteiro | -56.8908 | -19.0705 | 93 |
|  |  | Monteiro | -56.8908 | -19.0705 | 93 |
|  |  | Monteiro | -56.8282 | -18.9931 | 95 |
|  |  | Monteiro | -56.9261 | -18.8297 | 97 |
|  |  | Monteiro | -56.7746 | -18.9877 | 98 |
|  |  | Monteiro | -56.7746 | -18.9877 | 98 |
|  |  | Monteiro | -56.5962 | -19.2338 | 100 |
|  |  | Monteiro | -56.645 | -18.8833 | 102 |
|  |  | Monteiro | -56.6219 | -18.9864 | 102 |
|  |  | Monteiro | -56.6534 | -18.9314 | 104 |
|  |  | Monteiro | -56.6534 | -18.9314 | 104 |
|  |  | Monteiro | -56.4978 | -19.1059 | 107 |
|  |  | Monteiro | -56.5442 | -18.7826 | 107 |
|  |  | Monteiro | -56.6747 | -18.5731 | 109 |
| North | Pará | Marajoara | -48.6733 | -0.93897 | 2 |
|  |  | Marajoara | -48.6733 | -0.93897 | 2 |
|  |  | Marajoara | -48.6733 | -0.93897 | 2 |
|  |  | Marajoara | -48.6733 | -0.93897 | 2 |
|  |  | Marajoara | -48.6733 | -0.93897 | 2 |
|  |  | Marajoara | -48.6733 | -0.93897 | 2 |
|  |  | Marajoara | -48.6733 | -0.93897 | 2 |
|  |  | Marajoara | -48.6733 | -0.93897 | 2 |
|  |  | Marajoara | -48.6733 | -0.93897 | 2 |
|  |  | Marajoara | -48.6646 | -0.93397 | 3 |
|  |  | Marajoara | -48.6646 | -0.93397 | 3 |
|  |  | Marajoara | -48.6646 | -0.93397 | 3 |
|  |  | Marajoara | -48.6646 | -0.93397 | 3 |
|  |  | Marajoara | -48.6646 | -0.93397 | 3 |
|  |  | Marajoara | -48.6646 | -0.93397 | 3 |
|  |  | Marajoara | -48.6658 | -0.94303 | 4 |
|  |  | Marajoara | -48.6817 | -0.94053 | 4 |
|  |  | Marajoara | -48.6817 | -0.94053 | 4 |
|  |  | Marajoara | -48.6817 | -0.94053 | 4 |
|  |  | Marajoara | -48.6817 | -0.94053 | 4 |
|  |  | Marajoara | -48.6817 | -0.94053 | 4 |
|  |  | Marajoara | -48.6817 | -0.94053 | 4 |
|  |  | Marajoara | -48.6817 | -0.94053 | 4 |
|  |  | Marajoara | -48.6817 | -0.94053 | 4 |
|  |  | Marajoara | -48.6608 | -0.94494 | 4 |
|  |  | Marajoara | -48.6608 | -0.94494 | 4 |
|  |  | Marajoara | -48.6608 | -0.94494 | 4 |
|  |  | Marajoara | -48.6608 | -0.94494 | 4 |
|  |  | Marajoara | -48.6608 | -0.94494 | 4 |
| Notheast | Bahia | Piau | -40.3255 | -12.5358 | 260 |
|  |  | Piau | -40.3255 | -12.5358 | 260 |
|  |  | Piau | -40.3255 | -12.5358 | 260 |
|  | Paraíba | Crioulo | -35.6935 | -6.95953 | 557 |
|  |  | Crioulo | -35.6935 | -6.95953 | 557 |
|  | Pernambuco | Baé | -35.2933 | -8.00241 | 154 |
|  |  | Baé | -35.2933 | -8.00241 | 154 |
|  |  | Baé | -37.6667 | -8.70001 | 378 |
|  |  | Baé | -39.578 | -8.09019 | 384 |
|  |  | Baé | -38.2933 | -7.98413 | 438 |
|  |  | Baé | -39.1327 | -8.07315 | 445 |
|  |  | Baé | -39.1327 | -8.07315 | 445 |
|  |  | Baé | -40.0803 | -7.87701 | 450 |
|  |  | Baé | -40.0803 | -7.87701 | 450 |
|  |  | Baé | -37.2739 | -7.48048 | 581 |
|  |  | Canastrão | -39.1327 | -8.07315 | 445 |
|  |  | Canastrão | -40.0803 | -7.87701 | 450 |
|  |  | Canastrão | -37.54 | -7.58858 | 574 |
|  |  | Canastrão | -37.2739 | -7.48048 | 581 |
|  |  | Canastrão | -40.4968 | -7.57806 | 611 |
|  |  | Canastrão | -40.4968 | -7.57808 | 611 |
|  |  | Canastrão | -40.4968 | -7.57808 | 611 |
|  |  | Canastra | -39.578 | -8.09019 | 384 |
|  |  | Canastra | -39.578 | -8.09019 | 384 |
|  |  | Canastra | -39.6145 | -7.71214 | 437 |
|  |  | Canastra | -40.0803 | -7.87701 | 450 |
|  |  | Canastra | -39.9281 | -7.77058 | 451 |
|  |  | Canastra | -39.7093 | -7.51383 | 504 |
|  |  | Canastra | -37.2739 | -7.48048 | 581 |
|  |  | Canastra | -40.4968 | -7.57808 | 611 |
|  |  | Canastra | -39.578 | -8.09019 | 1047 |
|  |  | Caruncho | -39.6145 | -7.71214 | 384 |
|  |  | Caruncho | -39.6145 | -7.71214 | 437 |
|  |  | Caruncho | -39.1327 | -8.07315 | 437 |
|  |  | Caruncho | -48.013 | -15.9126 | 445 |
|  |  | Landrace | -34.9033 | -7.91016 | 26 |
|  |  | Landrace | -34.9415 | -7.85183 | 96 |
|  |  | Mammelado | -34.8723 | -7.94013 | 8 |
|  |  | Mestiço | -35.2603 | -8.09537 | 157 |
|  |  | Mestiço | -35.2603 | -8.09537 | 157 |
|  |  | Mestiço | -35.2603 | -8.09537 | 157 |
|  |  | Mestiço | -39.578 | -8.09019 | 384 |
|  |  | Mestiço | -40.0803 | -7.87701 | 450 |
|  |  | Moura | -39.578 | -8.09019 | 384 |
|  |  | Moura | -39.6145 | -7.71214 | 437 |
|  |  | Moura | -39.6145 | -7.71214 | 437 |
|  |  | Moura | -40.0803 | -7.87701 | 450 |
|  |  | Moura | -39.9281 | -7.77058 | 451 |
|  |  | Moura | -39.7093 | -7.51383 | 504 |
|  |  | Moura | -37.2739 | -7.48048 | 581 |
|  |  | Moura | -40.4968 | -7.57808 | 611 |
|  |  | Nilo | -39.578 | -8.09019 | 384 |
|  |  | Nilo | -39.1327 | -8.07315 | 445 |
|  |  | Nilo | -39.1327 | -8.07315 | 445 |
|  |  | Nilo | -39.7093 | -7.51383 | 504 |
|  |  | Nilo | -37.2739 | -7.48048 | 581 |
|  |  | Nilo | -40.4968 | -7.57808 | 611 |
|  |  | Nilo | -40.4968 | -7.57808 | 611 |
|  |  | Nilo | -39.6145 | -7.71214 | 1047 |
|  |  | Piau | -34.947 | -8.05197 | 13 |
|  |  | Piau | -35.1769 | -7.89192 | 77 |
|  |  | Piau | -35.2915 | -8.11393 | 157 |
|  |  | Piau | -39.578 | -8.09019 | 384 |
|  |  | Piau | -38.2933 | -7.98413 | 438 |
|  |  | Piau | -37.2739 | -7.48048 | 581 |
| South | Rio Grande do Sul | Caruncho | -52.6187 | -31.7283 | 106 |
|  |  | Caruncho | -52.6396 | -31.7305 | 158 |
|  |  | Caruncho | -52.6396 | -31.7305 | 158 |
|  |  | Mestiço | -52.6496 | -31.7307 | 155 |
|  |  | Moura | -52.5992 | -31.715 | 93 |
|  |  | Moura | -52.6454 | -31.7324 | 155 |
|  |  | Moura | -52.6454 | -31.7324 | 155 |
|  |  | Moura | -52.6496 | -31.7307 | 155 |
|  |  | Nilo | -52.0953 | -31.0771 | 74 |
|  |  | Nilo | -52.0953 | -31.0771 | 74 |
|  |  | Nilo | -52.1003 | -31.077 | 89 |
|  |  | Nilo | -52.1003 | -31.077 | 89 |
|  |  | Nilo | -52.5992 | -31.715 | 93 |
|  |  | Piau | -52.1003 | -31.077 | 89 |
|  | Santa Catarina | Casco de Burro | -49.0888 | -28.2109 | 401 |
|  |  | Duroc | -51.988 | -27.3129 | 586 |
|  |  | Duroc | -51.988 | -27.3129 | 586 |
|  |  | Duroc | -51.988 | -27.3129 | 586 |
|  |  | Duroc | -51.988 | -27.3129 | 586 |
|  |  | Landrace | -49.3076 | -28.1165 | 373 |
|  |  | Landrace | -49.3076 | -28.1165 | 373 |
|  |  | Landrace | -49.3076 | -28.1165 | 373 |
|  |  | Landrace | -49.3076 | -28.1165 | 373 |
|  |  | Landrace | -49.3076 | -28.1165 | 373 |
|  |  | Landrace | -51.988 | -27.3129 | 586 |
|  |  | Landrace | -51.988 | -27.3129 | 586 |
|  |  | Large White | -51.988 | -27.3129 | 586 |
|  |  | Large White | -51.988 | -27.3129 | 586 |
|  |  | Moura | -49.0002 | -28.1997 | 171 |
|  |  | Moura | -49.0002 | -28.1997 | 171 |
|  |  | Moura | -49.0888 | -28.2109 | 401 |
|  |  | Moura | -49.0888 | -28.2109 | 401 |
|  |  | Moura | -51.988 | -27.3129 | 586 |
|  |  | Moura | -51.988 | -27.3129 | 586 |
|  |  | Moura | -51.988 | -27.3129 | 586 |
|  |  | Moura | -51.988 | -27.3129 | 586 |
| Southeast | Minas Gerais | Piau | -42.8824 | -20.7543 | 658 |
|  |  | Pietran | -42.8608 | -20.7717 | 695 |
|  |  | Pietran | -42.8608 | -20.7717 | 695 |

Table S2: Environmental variables used in the identification of environmental influence in allele frequency of Brazilian locally adapted swine breeds (Geographic Data Base).

| Data Base | Environmental Variable | Data obtained | Data Calculated |
| --- | --- | --- | --- |
| Harvest Choise | Aridity | Ratio of precipitation to PET | - |
|  | PETannual | Annual potential evaporo-transpiration | - |
| World Clim | Temperature | Monthly maximum temperature | Means and medians from annual and seasonal periods |
|  |  | Monthly average temperature | Means and medians from annual and seasonal periods |
|  |  | Monthly minimum temperature | Means and medians from annual and seasonal periods |
|  | Solar Radiation | Monthly solar radiation | Means and medians from annual and seasonal periods |
|  | Precipitation | Monthly precipitation | Means and medians from annual and seasonal periods |
|  | Bioclimatic | Bio1-19 | - |
| Gtopo30 | Elevation | Elevation | - |

Data from last 50 years, resolution 30 arc.

PETannual=annual potential evaporo-transpiration; Aridity= ratio of precipitation to PET

Source: http://www.worldclim.org; https://lta.cr.usgs.gov/GTOPO30; [http://harvestchoice.org](http://harvestchoice.org/).

Table S3: AMOVA values among Brazilian regions.

| Source of variation | d.f. | Sum of squares | Variance components | Percentage of variation |
| --- | --- | --- | --- | --- |
| Among groups | 4 | 90740.541 | 46.30876Va | 0.87 |
| Among populations within groups | 5 | 57448.151 | 309.73284Vb | 5.79 |
| Within populations | 352 | 1759100.502 | 4997.44461Vc | 93.35 |
| Total | 361 | 1907289.193 | 5353.48620 |  |

Groups were formed with any animal sampled in a state within regions. Group 1: South Region (Santa Catarina and Rio Grande do Sul); Group 2: Midwest Region (Goiais, Mato Grosso and Mato Grosso do Sul); Group 3: Southeast Region (Minas Gerais); Group 4: Northeast Region (Bahia, Paraiba and Pernanbuco); Group 5 : North Region (Pará)

Vc; Vb: P(rand. value > obs. value) = 0.00000, P-value = 0.00000+-0.00000

Va: P(rand. value > obs. value) = 0.24772, P-value = 0.24772+-0.00443

Table S4: AMOVA values among commercial and Locally Adapted Brazilian Swine Genetic Groups

| Source of variation | d.f. | Sum of squares | Variance components | Percentage of variation |
| --- | --- | --- | --- | --- |
| Among groups | 1 | 26664.93 | 200.6518Va | 3.66 |
| Among populations within groups | 16 | 191515.1 | 340.3295Vb | 6.22 |
| Among individuals with in populations | 163 | 877994.2 | 452.5846Vc | 8.27 |
| Within individuals | 181 | 811115 | 4481.298Vd | 81.85 |
| Total | 361 | 1907289.193 | 5474.864 |  |

Group 1=Brazilian Locally adapted genetic groups (Bae, Canastra, Canastrao, Caruncho, Casco de Burro, Crioulo, Mammelado, Marajoara, Mestico, Monteiro, Moura, Nilo, Piau, Rabo de Peixe)

Group 2= Globally commercial breeds (Large White, Duroc, Pietran, Landrace)

Vd; Vc; Vb: P(rand. value &lt; obs. value) = 0.00000; P-value = 0.00000+-0.00000.

Va: P(rand. value > obs. value) = 0.00574; P-value = 0.00574+-0.00071

Table S5: F_IS_ Means, Minimum and Maximum values for Locally Adapted Brazilian Swine Genetic Groups and for States of Brazil.

|  | F_IS_ | | |
| --- | --- | --- | --- |
| Breed | Min | Average | Max |
| Bae | -0.042 | 0.198 | 0.428 |
| Canastra | -0.051 | 0.136 | 0.416 |
| Canastrao | -0.022 | 0.117 | 0.325 |
| Caruncho | 0.002 | 0.269 | 0.642 |
| CascodeBurro | 0.006 | 0.058 | 0.157 |
| Crioulo | -0.007 | -0.001 | 0.005 |
| Duroc | 0.188 | 0.208 | 0.241 |
| Landrace | -0.021 | 0.037 | 0.105 |
| LargeWhite | -0.004 | 0.059 | 0.116 |
| Mammelado | 0.009 | 0.009 | 0.009 |
| Marajoara | -0.019 | 0.097 | 0.348 |
| Mestico | -0.021 | 0.073 | 0.206 |
| Monteiro | 0.024 | 0.289 | 0.527 |
| Moura | -0.015 | 0.117 | 0.362 |
| Nilo | -0.050 | 0.178 | 0.586 |
| Piau | -0.042 | 0.175 | 0.565 |
| Pietran | 0.129 | 0.141 | 0.152 |
| RabodePeixe | 0.013 | 0.155 | 0.296 |
|  | F_IS_ | | |
| Estado | Min | Average | Max |
| GO | -0.028 | 0.196 | 0.630 |
| MS | 0.153 | 0.296 | 0.347 |
| MT | 0.025 | 0.043 | 0.072 |
| PA | -0.019 | 0.097 | 0.348 |
| PB | -0.007 | -0.001 | 0.005 |
| PE | -0.053 | 0.164 | 0.642 |
| RS | -0.025 | 0.070 | 0.251 |
| SC | -0.022 | 0.121 | 0.363 |
| MG | 0.098 | 0.249 | 0.361 |
| MG | 0.114 | 0.132 | 0.152 |

PA=Pará; BA=Bahia; PE=Pernambuco; PB=Paraíba; GO=Goiás; MS=Mato Grosso do Sul; MT=Mato Grosso; MG= Minas Gerais; RS=Rio Grande do Sul; SC=Santa Catarina.
